# Advanced fructo-oligosaccharides improve itching and aberrant epidermal lipid composition in children with atopic dermatitis

**DOI:** 10.3389/fmicb.2024.1383779

**Published:** 2024-04-29

**Authors:** Sukyung Kim, Bae-Gon Kang, Soonok Sa, Se Young Park, Kyungheon Ryu, Jinyoung Lee, Boram Park, Mijeong Kwon, Yeonghee Kim, Jiwon Kim, Sanghee Shin, Sehun Jang, Byung Eui Kim, Jaewoong Bae, Kangmo Ahn, Kwang-Hyeon Liu, Jihyun Kim

**Affiliations:** ^1^Department of Pediatrics, Samsung Medical Center, Sungkyunkwan University School of Medicine, Seoul, Republic of Korea; ^2^BK21 FOUR Community-Based Intelligent Novel Drug Discovery Education Unit, College of Pharmacy, Kyungpook National University, Daegu, Republic of Korea; ^3^Food R&D, Samyang Corporation, Seongnam, Republic of Korea; ^4^Biomedical Statistics Center, Research Institute for Future Medicine, Samsung Medical Center, Seoul, Republic of Korea; ^5^Department of Pediatrics, National Jewish Health, Denver, CO, United States; ^6^R&D Institute, BioEleven Co., Ltd., Seoul, Republic of Korea; ^7^Department of Health Sciences and Technology, Samsung Advanced Institute for Health Sciences and Technology, Seoul, Republic of Korea

**Keywords:** atopic dermatitis, fatty acid elongase, fructo-oligosaccharides, kestose, prebiotics

## Abstract

**Introduction:**

The effects of fructo-oligosaccharides (FOS) on atopic dermatitis (AD) have not been determined.

**Methods:**

In a randomized, double-blind, placebo-controlled trial, children with AD aged 24 months to 17 years received either advanced FOS containing 4.25 g of 1-kestose or a placebo (maltose) for 12 weeks.

**Results:**

The SCORAD and itching scores were reduced in patients treated with both FOS (all *p* < 0.01) and maltose (*p* < 0.05 and *p* < 0.01). Sleep disturbance was improved only in the FOS group (*p* < 0.01). The FOS group revealed a decreased proportion of linoleic acid (18:2) esterified omega-hydroxy-ceramides (EOS-CERs) with amide-linked shorter chain fatty acids (C28 and C30, all *p* < 0.05), along with an increased proportion of EOS-CERs with longer chain fatty acids (C32, *p* < 0.01).

**Discussion:**

FOS may be beneficial in alleviating itching and sleep disturbance, as well as improving skin barrier function in children with AD.

## 1 Introduction

Although the precise pathophysiology underlying atopic dermatitis (AD) remains incompletely understood, extensive studies have emphasized the significance of microbial dysbiosis and abnormalities in epidermal barrier proteins and lipids (Langan et al., 2020; Kim et al., 2023a,b). Recent investigations suggest that oral administration of prebiotics, including galactooligosaccharides (GOS) and fructo-oligosaccharides (FOS) hold promise in treating AD by serving as energy sources for gut microbes and generating short-chain fatty acids that enhance the growth of beneficial microorganisms (Kim et al., 2021; Kim S. et al., 2022; Ahn, 2023). Among FOS variants, 1-kestose (GF2), a trisaccharide formed by the combination of a fructose molecule with glucose, can lower pH in culture media and stimulate the growth of *Bifidobacterium breve*, *B. longum*, *B. bifidum*, and *B. adolescentis* more effectively than other variants (Tochio et al., 2018). While normal FOS, which is composed of 35% 1-kestose, is used widely in food products, dietary supplements, animal feed, and pharmaceuticals, kestose-enriched advanced FOS (>85% 1-kestose) has been developed as the dominant component in synthetic FOS products (Kim et al., 2021).

Despite the strong prebiotic effect of purified 1-kestose at lower doses, as well as fewer adverse reactions such as diarrhea, abdominal distention, and flatulence (Tochio et al., 2018), its therapeutic effect on AD has been investigated in few studies. Koga et al. (2016) who conducted a randomized controlled trial of 1-kestose for 6 weeks, found a significant positive correlation between increased counts of gut *Faecalibacterium prausnitzii* and improvements in SCORing Atopic Dermatitis (SCORAD) scores in children aged 2 to 5 years with AD. Similarly, Shibata et al. (2009) revealed lower SCORAD scores in AD patients who received 2 g of 1-kestose daily for 12 weeks compared to the placebo group in a randomized, double-blind, placebo-controlled study involving children under 3 years of age. Despite the potential beneficial effects of 1-kestose in AD treatment, previous studies lack substantial evidence due to limitations such as insufficient clinical improvement and the absence of mechanism studies (Ahn, 2023). Moreover, in Shibata et al.’s study, no significant correlation was found between improvements in SCORAD score and *Bifidobacterium* counts.

We previously reported the beneficial effects of GOS on skin-barrier function and AD-like skin symptoms, suggesting the possibility of direct effects from prebiotics or probiotics on AD skin (Kim S. et al., 2022). However, mechanisms that explain the direct and indirect effects of 1-kestose on human AD skin have yet to be identified. Therefore, we aimed to examine the impacts of 1-kestose on AD skin beyond the clinical aspects, including the modulation of epidermal skin barrier and lipid profiles as well as skin microbiome.

## 2 Materials and methods

### 2.1 Study population and design

In this randomized, double-blind, placebo-controlled study, we enrolled patients aged 2–17 years diagnosed with AD based on the criteria established by Hanifin and Rajka (1980), with SCORAD scores ranging from 15 to 50. Exclusion criteria were recent administration of antibiotics, systemic corticosteroids, or phototherapy within the past 4 weeks; use of systemic immunosuppressants or biologics within the previous 4 weeks; intake of probiotics, prebiotics, or herbal medicine within the past week; use of antihistamines within the previous 2 weeks; and concurrent presence of other skin diseases or systemic illnesses.

Patients were randomly assigned to receive either advanced FOS or placebo treatments in a double-blind manner, using a computer-generated random-number list. An investigator not involved in the trial prepared the random-number list, which was then posted to the bags containing the materials. Samples of advanced FOS (composed of >85% 1-kestose) and maltose powder were provided by Food R&D, Samyang Corp (Seongnam, Korea). Over the course of 12 weeks, patients were orally administered either advanced FOS at a dosage of 2.5 g twice daily or a placebo of identical appearance and taste. During the study period, emollients (Atobarrier lotion MD, Aestura, Seoul, Korea) and topical corticosteroids (TCS; hydrocortisone 1% and prednisolone valeroacetate 0.3%) were made available during the study. To quantitatively assess their usage, the tubes containing emollients and TCS were weighed at each visit. The study was approved by the Institutional Review Board of Samsung Medical Center in Seoul, and written informed consent was obtained from parents prior to participation in the study (SMC IRB file No. 2020-11-155). Our study protocol was registered in the WHO ICTRP (International Clinical Trials Registry Platform). The registration number is KCT0006816.

### 2.2 Clinical evaluation and laboratory tests

**Figure 1:**
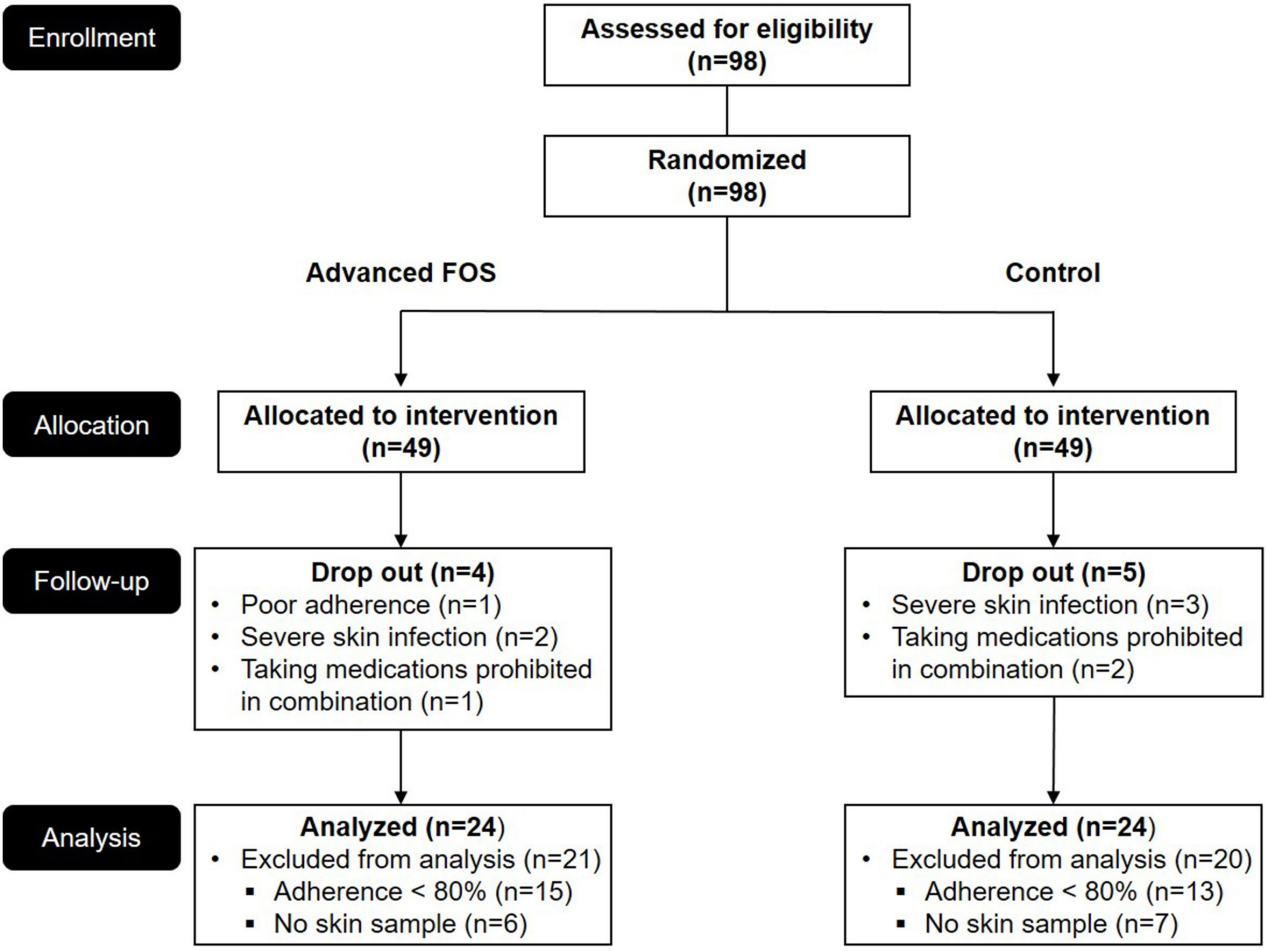
CONSORT diagram of this study. FOS, fructo-oligosaccharide.

Before initiation of treatment (T1) using either advanced FOS or placebo and upon completion of treatment (T2), children underwent clinical evaluations and blood tests (Figure 1). The severity of AD was assessed using the SCORAD score, ranging from 0 to 103 (European Task Force on Atopic Dermatitis, 1993). This grading system includes objective and subjective scores, evaluating pruritus and sleep disturbance on scales from 0 to 10. Measurements were performed for eosinophil counts in the peripheral blood, mRNA expression levels of fatty-acid elongation enzymes, and serum total immunoglobin E (IgE) levels determined by immunoCAP tests (ThermoFisher Scientific Inc., Waltham, MA). Skin tape stripping and skin microbiome samples were collected at T1 and T2.

### 2.3 Bacterial 16S rRNA sequencing of skin samples

Hypervariable regions V3-V4 of the 16S rRNA bacterial gene were amplified on skin samples collected and sequenced with an Illumina MiSeq platform (Illumina, San Diego, CA, USA). The 16S rRNA gene sequence data were analyzed using Quantitative Insights Into Microbial Ecology software (v1.9.1; Caporaso et al., 2010). Using qualified sequences (Phred ≥ Q20), operational taxonomic units were identified and quantified using an open reference method that mapped sequences with 97% identity to known sequences in the Greengenes database (v13.8) using UCLUST alignment algorithms and the EzBioCloud database^1^ (DeSantis et al., 2006; Edgar, 2010; Yoon et al., 2017).

### 2.4 Preparation of human stratum corneum and analysis of the lipid profiles

Samples of human stratum corneum (SC) were collected from the right forearm skin using D-squame tape with a diameter of 22 mm (CuDerm, Dallas, TX). Four consecutive D-squame strips were collected from each subject. The first tape disk was discarded, and the remaining three were placed in separate Eppendorf tubes (1.5 mL). Skin ceramides were extracted from the tape-stripping samples following a modified Sadowski method (Kim B. K. et al., 2022). In brief, each tape was vortexed with 1 mL of methanol for 1 h and centrifuged at 14,000 g and 4°C for 15 min. After removing the tape and debris, the extracts from three consecutive tape strips were pooled and dried under a nitrogen stream. The dried extracts were reconstituted in 100 μL of a chloroform-methanol mixture (1:9, *v/v*) containing the internal standard of EO-type ceramide (EOS, d18:1/26:0/ d9-18:1) purchased from Avanti Polar Lipids (Alabaster, AL, United States). Targeted skin lipidome analysis was performed using a modified Shimadzu LCMS-8060 liquid chromatograph–mass spectrometer (Shimadzu Corporations, Kyoto, Japan; Chu et al., 2023). Skin lipids were separated on a reversed-phase Kinetex C18 column (100 × 2.1 mm, 2.6 μm; Phenomenex, Torrance, CA, USA). Mobile phases A and B consisted of 10 mM ammonium acetate in water/methanol (1/9, *v/v*) and 10 mM ammonium acetate in methanol/isopropanol (1/1, *v/v*), respectively. The gradient elution was as follows: 0 min (30% B), 0–15 min (95% B), 15–20 min (95% B), and 20–25 min (30% B). The flow rate was 0.2 mL/min. Quantitation was performed using selected reaction monitoring of the [M + H]^+^ ion and the related product ion for each lipid. Each lipid was quantified as the peak area ratio of the target analyte to that of the internal standard multiplied by the concentration of the standard (Suzuki et al., 2022).

### 2.5 Human primary keratinocytes culture and real-time PCR

Human primary keratinocytes (HEKs; Thermo Fisher Scientific, Walthamm, MA, Cat #: C0015C) were derived from neonatal foreskin and grown in serum-free EpiLife™ cell culture medium (Life Technologies, Grand Island, NY, United States) containing 0.06 mmol/L calcium chloride, 1% human keratinocyte growth supplement S7 (Life Technologies), and 1% gentamicin/amphotericin as described previously (Kim S. et al., 2022). To investigate the effects of the advanced FOS on gene expression of fatty acid elongases (*ELOV*Ls) 1–6, ceramide synthase (*CERS*) 2 and 3, filaggrin (*FLG*), loricrin (*LOR*), and involucrin (*IVL*), HEKs were differentiated in the presence of CaCl_2_ (1.3 mmol/L) for 3 days. Cells were then incubated with maltose (1.0%), advanced FOS (1.0%), or a combination, interleukin (IL) 4 (5 ng/mL, R&D Systems, Minneapolis, MN, United States) and IL-13 (5 ng/mL, R&D Systems) for an additional 24 h. All reagents, including maltose, advanced FOS, and cytokines, were diluted using serum-free EpiLife medium for *in vitro* experiments.

RNeasy Mini Kits (Qiagen, Valencia, CA, USA) were used according to the manufacturer’s protocol to isolate RNA from keratinocytes. The RNA was reverse-transcribed into cDNA using a SuperScript VILO™ MasterMix according to the manufacturer’s protocol (Life Technologies). The cDNA was analyzed using real-time quantitative polymerase chain reaction (PCR) amplification and an ABI prism 7900 real-time PCR instrument (Applied Biosystems, Foster City, CA, United States) as described previously (Kim S. et al., 2022). Primers and probes for ELOVL1-6, CERS2, CERS3, FLG, LOR, IVL, and 18 s were purchased from Applied Biosystems and normalized to the 18 s mRNA levels.

### 2.6 Statistical analysis

The sample size was determined based on previous studies (Han et al., 2012; Jeong et al., 2020), estimating a difference of 6.5 in SCORAD change scores between the FOS and placebo groups. We calculated that 49 patients in each group (80% power, 5% significance, with a 20% dropout rate) would be needed. The final analysis included patients with adherence exceeding 80%, and skin samples were collected at both T1 and T2. All statistical analyses were conducted using GraphPad Prism, version 9 (San Diego, CA, United States) and SAS® (version 9.4, SAS Institute, Cary, NC, United States). Chi-squared test or Fisher’s exact test was performed to explore the difference for categorical data between the FOS and the control groups. For comparisons between the groups, independent t-test or Wilcoxon signed-rank test was used. For comparisons between T1 and T2 time points within group, paired t-test or Wilcoxon rank-sum test was applied. Statistical differences between 3 or more groups were determined using a one-way ANOVA, and significant differences were determined by a Tukey–Kramer post-hoc test. Additionally, Spearman’s correlation was used to evaluate correlations between variables. Error bars represent the SEM, and significance was set at a *p* value < 0.05.

## 3 Results

### 3.1 Advanced FOS improved atopic dermatitis symptoms

**Figure 2:**
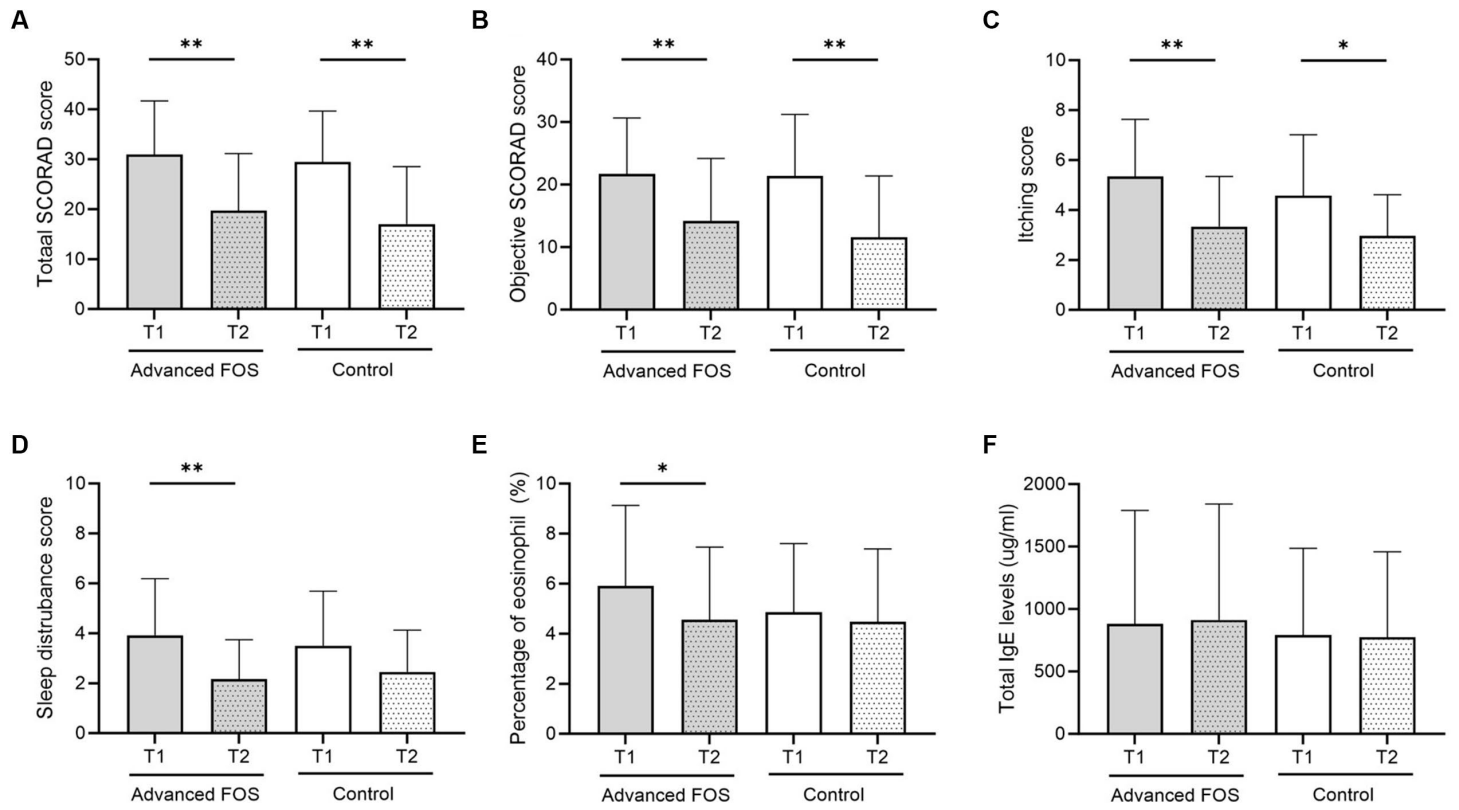
Changes in total **(A)** and objective SCORAD **(B)** scores, itching scores **(C)**, sleep disturbance scores **(D)**, peripheral blood eosinophil percentages **(E)**, and total IgE levels **(F)** at T2 compared to baseline (T1) in the FOS and control groups. ^*^p < 0.05, ^**^p < 0.01. FOS, fructo-oligosaccharide; IgE, immunoglobulin E; SCORAD, SCORing Atopic Dermatitis.

Analysis was conducted on 48 patients (24 in each group), all of whom had adherence exceeding 80% and provided skin samples (Figure 1). There were no differences in sex, age, comorbid allergic diseases, family history of allergic diseases, baseline SCORAD scores, the level of total IgE and the eosinophil count between the two groups (Table 1). The total scores and the objective scores of the SCORAD at T2 were significantly decreased in both groups (all *p* < 0.01; Figures 2A,B). The itching scores were reduced in both the FOS and the control groups, but showed a more significant decrease in the FOS group compared to the control group (*p* = 0.001 vs. *p* = 0.043; Figure 2C). Sleep disturbance scores were significantly decreased only in the FOS group at T2 (*p* = 0.003), while no changes were found in the control group (*p* = 0.134; Figure 2D). In the FOS group, the percentage of eosinophil was significantly lower at T2 than T1 (*p* = 0.024), whereas no significant changes were observed in the control group (*p* = 0.413; Figure 2E). However, there were no significant changes in the level of total IgE in either group between T1 and T2 (Figure 2F). Similarly, there were no significant differences in the amount of emollients and topical corticosteroids used during the study period (*p* = 0.802 and 0.209, respectively). No serious adverse events occurred in either group, and only 1 patient in the control group dropped out due to adverse events. No adverse events related to the intervention were reported in either group.

**Table 1:** Baseline clinical characteristics of subjects.

Variables	FOS group (*n* = 24)	Control group (*n* = 24)	*P-*value
Sex (male, *n*)	15 (62.5%)	15 (62.5%)	1.000
Age (year)	8.29 ± 3.51	8.54 ± 4.30	0.950
Age at diagnosis of atopic dermatitis (year)	3.38 ± 3.74	2.63 ± 3.42	0.397
Comorbid conditions	17 (70.8%)	18 (75.0%)	1.000
Food allergy	9 (37.5%)	9 (37.5%)	1.000
Asthma	2 (8.3%)	3 (12.5%)	1.000
Allergic rhinitis	9 (37.5%)	10 (41.7%)	1.000
Allergic conjunctivitis	2 (8.3%)	1 (4.2%)	1.000
Family history of allergic diseases	15 (62.5%)	17 (70.8%)	0.760
SCORAD	30.98 ± 10.71	29.42 ± 10.24	0.613
Objective score	21.73 ± 8.91	21.33 ± 9.89	0.657
Subjective score	9.25 ± 4.07	8.08 ± 4.26	0.335
Itching	5.33 ± 2.30	4.58 ± 2.43	0.242
Sleep disturbance	3.92 ± 2.26	3.50 ± 2.19	0.312
Eosinophil count (/mm^3^)	388.06 ± 254.65	330.95 ± 213.52	0.495
Total IgE (kU/L)	881.39 ± 908.38	791.29 ± 694.51	0.901

Data were represented as mean ± standard deviation or frequency (proportion).FOS, fructo-oligosaccharide; Ig, immunoglobulin; IGA, Investigator Global Assessment; SCORAD, SCORing Atopic Dermatitis.

### 3.2 Advanced FOS decreased abundance of *Lachnospiraceae* and increased abundance of *Prevotella* in the AD skin

**Figure 3:**
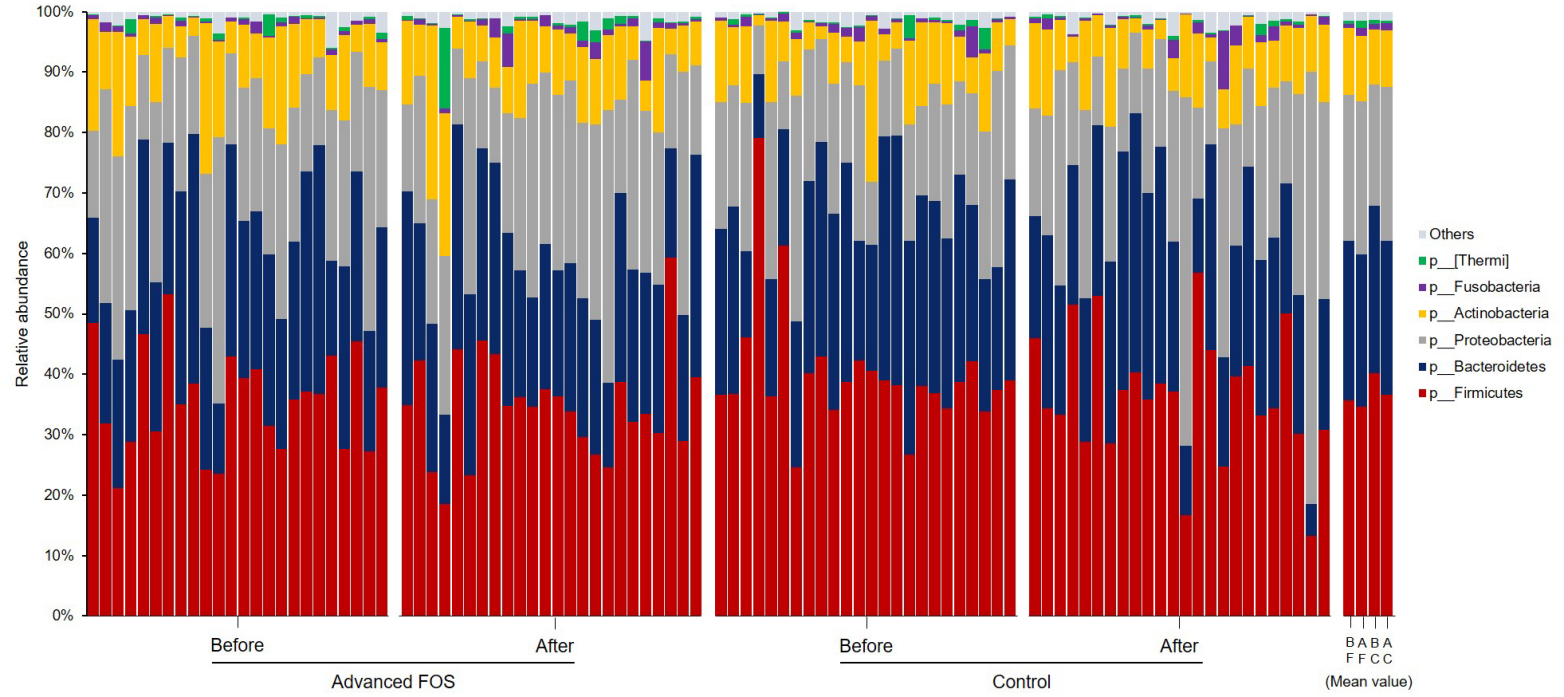
Skin microbiome composition at the phylum levels in the FOS and control groups. FOS, fructo-oligosaccharide; B, before; A, after, F, FOS group; C, control group.

**Figure 4:**
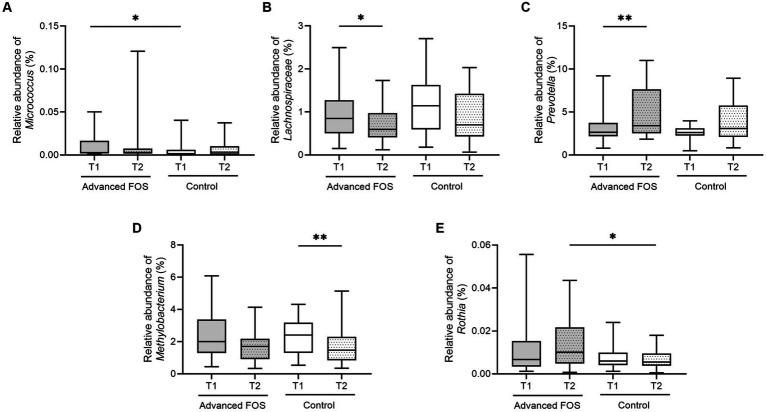
The change in relative abundance of *Micrococcus* **(A)**, *Lachnospiraceae* **(B)**, *Prevotella* **(C)**, *Methylobacterium* **(D)**, and *Rothia* **(E)** in the FOS and control groups. *p < 0.05, **p < 0.01. FOS, fructo-oligosaccharide.

No significant differences were observed in the bacterial phyla between the FOS and the control group both at T1 and T2 (all *p* > 0.05; Figure 3). However, at the genus level, a higher abundance of *Micrococcus* was found in the FOS group compared to the control group at T1 (*p* = 0.049; Figure 4A). After 12 weeks of treatment, the FOS group showed decreased abundance of *Lachnospiraceae* (*p* = 0.040; Figure 4B) and increased abundance of *Prevotella* (*p* = 0.007; Figure 4C), while the control group revealed a decreased abundance of *Methylobacterium* (*p* = 0.008; Figure 4D). Additionally, a higher abundance of *Rothia* was noted in the FOS group compared to the control group at T2 (*p* = 0.025; Figure 4E).

### 3.3 Advanced FOS altered the proportion of EOS-CERs with shorter and longer chain fatty acids in the AD skin

**Figure 5:**
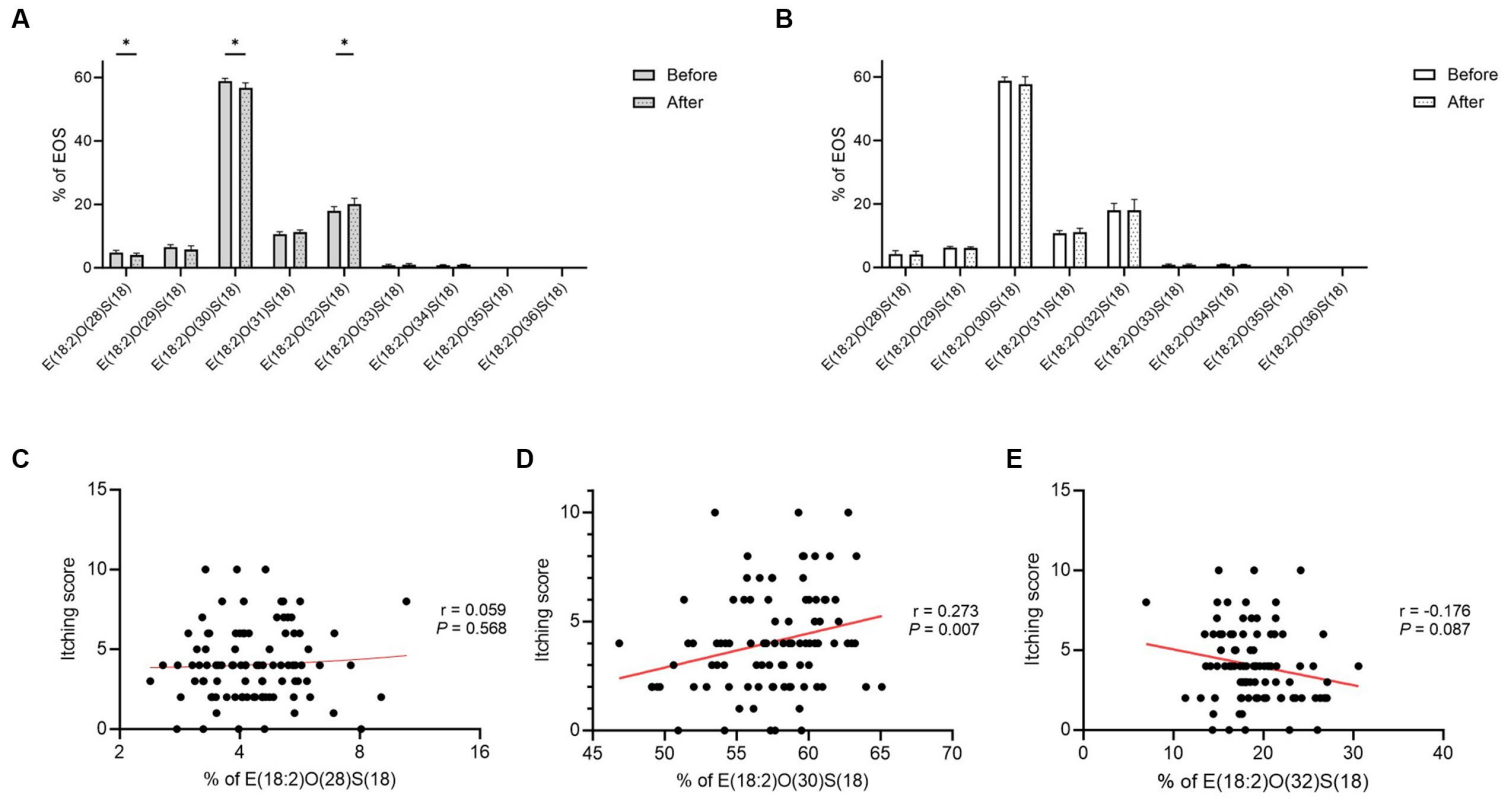
Proportion of EOS-CERs in the FOS **(A)** and control **(B)** groups and correlation between itching scores and the proportion of EOS-CERs with amide-linked C28 **(C)**, C30 **(D)**, and C32 **(E)** fatty acids in all subjects. ^*^p < 0.05, ^**^p < 0.01. EOS-CER, linoleic acid (18:2) esterified omega-hydroxy-ceramide; FOS, fructo-oligosaccharide.

The proportion of linoleic acid (18:2) esterified omega-hydroxy-ceramides (EOS-CERs) with amide-linked shorter chain fatty acids (C28 and C30) was decreased significantly in the FOS group (*p =* 0.029 and 0.013; Figure 5A). Additionally, the proportion of EOS-CERs with amide-linked longer chain fatty acids (C32) was increased in the FOS group (*p* = 0.010; Figure 5A). However, no significant change was observed in the proportion of EOS-CERs in the control group (Figure 5B). While no relationship between itching scores and EOS-CERs with amide-linked C28 fatty acids (*p* = 0.568), itching scores were positively correlated with EOS-CERs with amide-linked C30 fatty acids (*p* = 0.007; Figures 5C,D). The negative relationship between itching scores and the proportion of EOS-CERs with amide-linked longer-chain fatty acids (C32) was not statistically significant (*p =* 0.087; Figure 5E).

### 3.4 Advanced FOS increased expression of fatty acid elongase3 and filaggrin in cultured human primary keratinocytes

**Figure 6:**
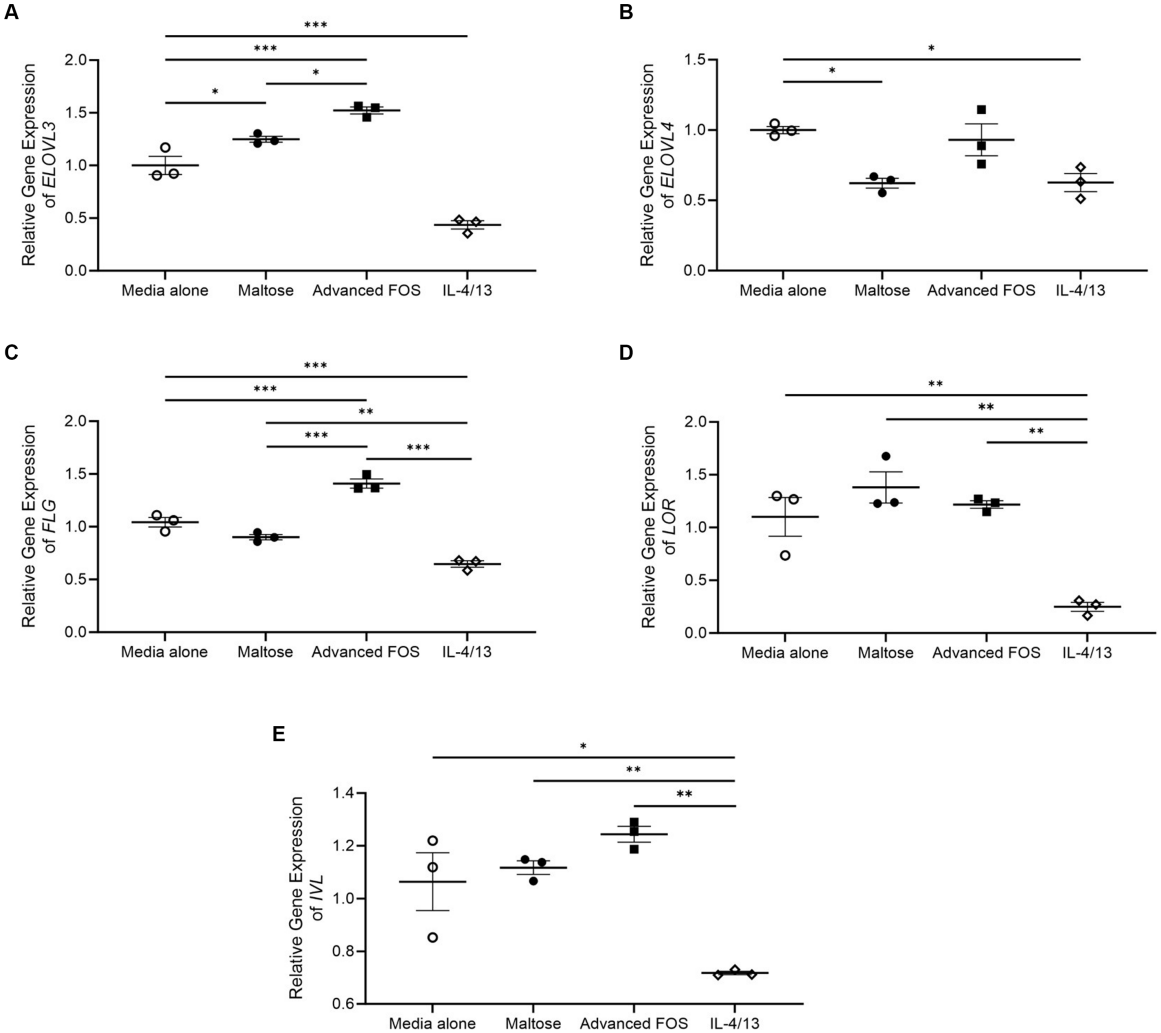
Gene expression of *ELOVL3* **(A)**, *ELOVL4* **(B)**, *FLG* **(C)**, *LOR* **(D)**, and *IVL* **(E)** in primary epidermal keratinocytes treated with media alone, maltose, advanced FOS, or a combination of IL-4 and IL-13. *p < 0.05, **p < 0.01, ***p < 0.001. ELOVL, elongating of very long chain fatty acids; FLG, filaggrin; FOS, fructo-oligosaccharide; IVL, involucrin; LOR, loricrin.

Gene expression of *ELOVL3* was significantly upregulated in HEKs treated with advanced FOS compared to those treated with media (*p* < 0.001) or maltose (*p* = 0.025; Figure 6A). Gene expression of *ELOVL3* was significantly decreased by a combination of IL-4 and IL-13 as expected (*p* < 0.001; Figure 6A; Berdyshev et al., 2018). Gene expression of *ELOVL4* was not affected by advanced FOS (Figure 6B). Likewise, gene expression of *ELOVL1*, *2*, *5*, *6*, *CERS2*, and *CERS3* was not affected by advanced FOS (Figure S1). Additionally, gene expression of *ELOVL3* was significantly increased in HEKs treated with maltose compared to those treated with media alone (*p* = 0.039; Figure 6A). However, gene expression of *ELOVL4* was decreased by maltose (*p* = 0.020; Figure 6B).

Moreover, gene expression of *FLG* and was significantly upregulated in HEKs treated with advanced FOS compared to those treated with media alone (*p* < 0.001) or maltose (*p* < 0.001; Figure 6C). Gene expression of *FLG* was significantly decreased by a combination of IL-4 and IL-13 as expected (Figure 6C; Howell et al., 2009). However, gene expression of *LOR* (Figure 6D) and *IVL* (Figure 6E) was not affected by maltose or advanced FOS.

## 4 Discussion and conclusion

In the present study, we showed that advanced FOS can alleviate symptoms and be safely consumed in children with AD. Over a 12-week treatment period, the FOS group displayed a more pronounced decrease in itching scores and sleep disturbance compared to the control group. Furthermore, the FOS group exhibited a decrease in eosinophil counts and specific alterations in the skin microbiome and epidermal lipid profiles. These findings suggest potential mechanisms underlying the observed clinical improvements.

Itching, a characteristic symptom of AD, triggers scratching, exacerbating skin lesions, and perpetuating a detrimental cycle (Langan et al., 2020). This cycle detrimentally impacts sleep quality, thereby affecting the overall well-being of children with AD and their families (Ständer et al., 2019). The substantial improvement in itching and sleep disturbances observed with FOS treatment was clinically relevant in our study, potentially preventing vicious cycles and chronicity in children with AD and positively influencing their quality of life. These clinical improvements correlate with changes in the percentage of eosinophils in peripheral blood, indicating that immunologic alteration contributes to the therapeutic effect of the prebiotics. These results correspond with those of earlier studies which reported significant improvements in itching and sleep disturbances following a 12-week intake of advanced FOS in children under 3 years of age with AD (Shibata et al., 2009). However, unlike our study, that study showed a reduction in SCORAD scores, while both the total and objective SCORAD scores were reduced in both the FOS and control groups in our current study. Discrepancies in host age, AD severity, ethnicity, or atopic characteristics might account for these differences. It is also possible that the effects observed in the control group may be attributed to the effects of maltose.

Skin barrier dysfunction is characteristic finding of AD, substantially contributing to its progression and the manifestation of pruritic symptoms (Kim and Leung, 2018; Leung et al., 2020; van den Bogaard et al., 2023). Therefore, to understand the mechanisms underlying the positive effects of advanced FOS on AD symptoms, we investigated whether advanced FOS modulates the expression of *ELOVL*s and epidermal barrier proteins, which are associated with skin barrier function (Kim and Leung, 2018). We found that advanced FOS increased expression of *ELOVL3*, which is a key enzyme for elongation of C16-C22 fatty acids (Zwara et al., 2021). It has been reported that *ELOVL3*-ablated mice exhibit a defect in water repulsions and increased transepidermal water loss (TEWL; Westerberg et al., 2004). Additionally, we demonstrated that advanced FOS upregulated the gene expression of *FLG*, a key epidermal barrier protein maintaining skin barrier function (Irvine et al., 2011; Kim and Leung, 2018). Advanced FOS may improve AD symptoms by upregulating *ELOVL3* and *FLG*, which play important roles in skin barrier function, despite the absence of significant alterations in TEWL following administration of advanced FOS (data not shown). However, our study did not detect notable alterations in TEWL following administration of advanced FOS, suggesting that factors other than *FLG* and *ELOVL3* contribute to TEWL changes.

Omega-O-acylceramides (AcylCERs), specific skin ceramides characterized by ultralong N-acyl chains esterified with linoleic acid, constitute 7%–12% of human SC ceramides and indispensable for the maintenance of skin barrier function (Opálka et al., 2016). Their scarcity, reduced by 15%–50% in AD patients compared to healthy individuals, significantly impacts skin barrier integrity (Janssens et al., 2012). Biosynthesis of EOS-CERs, one of the most abundant AcylCERs, is disrupted in AD keratinocytes, leading to the disorganization of formed lamellae and a disrupted skin barrier (Leung et al., 2020). Furthermore, the impact of fatty acid chain length in epidermal lipids on membrane structure and permeability emphasizes the pivotal role of disrupted skin barriers associated with irregular lipid layers in AD pathogenesis (Knox and O'Boyle, 2021). CERs containing shorter chain fatty acids rarely interact with other lipid chains, affecting barrier functions despite similar polar head groups and the ability to form hydrogen bonds with longer chain fatty acids (Ishikawa et al., 2010). In this study, proportion of EOS-CER having C28 and C30 acyl chains was significantly decreased in the FOS group, while EOS-CER with C32 acyl chain was significantly increased. However, it remains unclear how oral administration of prebiotics influences skin lipid profiles through the enzymatic pathway.

Previous studies showed an increased presence of *Staphylococcus* and reduced abundance of *Corynebacterium* and *Micrococcus* in AD skin compared to healthy skin (Kim et al., 2023b). Levels of long-chain unsaturated free fatty acids (FFAs) in CERs are reportedly associated with increases in the abundance of *Propionibacteria* and *Corynebacterium* in the skin of pediatric and adult AD patients, suggesting a potential interplay between SC lipids and these microbes (Baurecht et al., 2018; Kim et al., 2023b). Similarly, our study revealed changes in both SC lipid profiles and skin microbiome subsequent to the administration of advanced FOS in children with AD, although the exact sequence of these changes remains unclear. *Prevotella,* has been reported to exhibit a negative correlation with the severity of AD after topical corticosteroid treatment (Zheng et al., 2019). In infants with AD aged 0–1 year, the relative abundance of *Rothia* declined significantly with increased AD severity in the perioral skin area (Zheng et al., 2019). Our observation of an increase in *Rothia* abundance after 12 weeks in the FOS group suggests that kestose induces an improvement in AD by upregulating the abundance of beneficial microbes. Chng et al. reported a lower abundance of *Methylobacterium* in AD skin compared to healthy skin, and these bacteria showed a negative correlation with *S. aureus* (Chng et al., 2016; Olesen et al., 2018). In our study, *Methylobacterium* abundance tended to decrease in both groups after 12 weeks, with a more pronounced decrease in the control group compared to the FOS group. This reduction could be attributed to the potential impact of advanced FOS on *Methylobacterium* reduction, recognized as beneficial bacteria, or may be associated with *S. aureus* distribution, necessitating further investigation. In addition, most previous studies were based on the distribution of *Lachnospiraceae* in feces, and there is little research on the relationship between the distribution of *Lachnospiraceae* in the skin and AD. Results of animal experiments using dogs with allergies showed that the relative abundance of *Lachnospiraceae* on skin had a positive correlation with the percentage of CD4 + CD25 + Foxp3+ Treg cells (Rostaher et al., 2021). However, unlike the animal test results, the abundance of *Lachnospiraceae* was lower even when the skin condition improved after 12 weeks in our study. This suggests that the action of *Lachnospiraceae* may vary depending on its distribution, and that its effects differ by disease; additional research is warranted.

This study has limitations arising from the sample size and the selection of the placebo. The smaller-than-anticipated sample size may have limited our ability to detect any clinical effects of advanced FOS. Our findings revealed that maltose, which is commonly used as a placebo in randomized controlled trials assessing prebiotics, resulted in an upregulation of the expression of *ELOVL3*, which is a key player in skin lipid elongation, similar to kestose (Passeron et al., 2006; Shibata et al., 2009; Koga et al., 2016; Kim et al., 2021). However, in the current study, maltose increased the gene expression of *ELOVL 3* like kestose, but the effect is found to be less than that of kestose. Additionally, unlike kestose, maltose reduced the gene expression of *ELOVL 4*, a key player in very long-chain fatty acid production (Cameron et al., 2007). This finding suggests that kestose may have greater positive effects on skin lipids compared to maltose. In the case of skin barrier proteins in the current study, maltose upregulated the gene expression of LOR in the current study, which is crosslinked to IVL, envoplakin, and periplakin scaffolds, thereby reinforcing the cornified envelope (Furue, 2020). Further investigation is necessary to elucidate determine whether maltose genuinely enhances skin barrier function. Nevertheless, advanced FOS showed had a favorable impact on alleviating itching and sleep disturbance, indicating its potential influence not only in microbial communities of the skin, but also on SC lipids responsible for maintaining skin barrier integrity.

In conclusion, the oral administration of advanced FOS demonstrated both safety and beneficial effects in itching and sleep disturbance while inducing specific alterations in the skin microbiome and epidermal lipid profiles among children with AD throughout a 12-week treatment period. This suggests that advanced FOS may be beneficial in improving skin symptoms and enhancing skin barrier function in children with AD.

## Data availability statement

The authors acknowledge that the data presented in this study must be deposited and made publicly available in an acceptable repository, prior to publication. Frontiers cannot accept a manuscript that does not adhere to our open data policies.

## Ethics statement

The studies involving humans were approved by the Institutional Review Board of Samsung Medical Center in Seoul (SMC IRB file No. 2020-11-155). The studies were conducted in accordance with the local legislation and institutional requirements. Written informed consent for participation in this study was provided by the participants’ legal guardians/next of kin.

## Author contributions

SK: Conceptualization, Data curation, Formal analysis, Methodology, Visualization, Writing – original draft. B-GK: Formal analysis, Writing – original draft. SSa: Funding acquisition, Writing – review & editing. SP: Funding acquisition, Writing – review & editing. KR: Funding acquisition, Writing – review & editing. JL: Formal analysis, Writing – review & editing. BP: Formal analysis, Methodology, Validation, Writing – review & editing. MK: Data curation, Writing – review & editing. YK: Data curation, Writing – review & editing. JiwK: Data curation, Writing – review & editing. SSh: Data curation, Writing – review & editing. SJ: Data curation, Writing – review & editing. BK: Methodology, Writing – review & editing. JB: Formal analysis, Software, Visualization, Writing – review & editing. KA: Writing – review & editing. K-HL: Validation, Writing – original draft, Writing – review & editing. JihK: Conceptualization, Methodology, Project administration, Software, Supervision, Validation, Writing – original draft, Writing – review & editing.

## Footnotes

^1^ http://www.ezbiocloud.net

## References

[ref1] Ahn K. (2023). The effect of prebiotics on atopic dermatitis. Allergy Asthma Immunol Res. 15, 271–275. doi: 10.4168/aair.2023.15.3.271, PMID: 37188483 PMC10186120

[ref2] Baurecht H. Ruhlemann M. C. Rodriguez E. Thielking F. Harder I. Erkens A. S.. (2018). Epidermal lipid composition, barrier integrity, and eczematous inflammation are associated with skin microbiome configuration. J. Allergy Clin. Immunol. 141, 1668–1676.e16. doi: 10.1016/j.jaci.2018.01.019, PMID: 29421277

[ref3] Berdyshev E. Goleva E. Bronova I. Dyjack N. Rios C. Jung J.. (2018). Lipid abnormalities in atopic skin are driven by type 2 cytokines. JCI Insight 3:e98006. doi: 10.1172/jci.insight.98006, PMID: 29467325 PMC5916244

[ref4] Cameron D. J. Tong Z. Yang Z. Kaminoh J. Kamiyah S. Chen H.. (2007). Essential role of Elovl4 in very long chain fatty acid synthesis, skin permeability barrier function, and neonatal survival. Int. J. Biol. Sci. 3, 111–119. doi: 10.7150/ijbs.3.111, PMID: 17304340 PMC1796949

[ref5] Caporaso J. G. Kuczynski J. Stombaugh J. Bittinger K. Bushman F. D. Costello E. K.. (2010). QIIME allows analysis of high-throughput community sequencing data. Nat. Methods 7, 335–336. doi: 10.1038/nmeth.f.303, PMID: 20383131 PMC3156573

[ref6] Chng K. R. Tay A. S. Li C. Ng A. H. Wang J. Suri B. K.. (2016). Whole metagenome profiling reveals skin microbiome-dependent susceptibility to atopic dermatitis flare. Nat. Microbiol. 1:16106. doi: 10.1038/nmicrobiol.2016.106, PMID: 27562258

[ref7] Chu H. Kim S. M. Zhang K. Wu Z. Lee H. Kim J. H.. (2023). Head and neck dermatitis is exacerbated by Malassezia furfur colonization, skin barrier disruption, and immune dysregulation. Front. Immunol. 14:1114321. doi: 10.3389/fimmu.2023.1114321, PMID: 36911720 PMC9992991

[ref8] DeSantis T. Z. Hugenholtz P. Larsen N. Rojas M. Brodie E. L. Keller K.. (2006). Greengenes, a chimera-checked 16S rRNA gene database and workbench compatible with ARB. Appl. Environ. Microbiol. 72, 5069–5072. doi: 10.1128/AEM.03006-05, PMID: 16820507 PMC1489311

[ref9] Edgar R. C. (2010). Search and clustering orders of magnitude faster than BLAST. Bioinformatics 26, 2460–2461. doi: 10.1093/bioinformatics/btq461, PMID: 20709691

[ref10] European Task Force on Atopic Dermatitis (1993). Severity scoring of atopic dermatitis: the SCORAD index. Consensus report of the European task force on atopic dermatitis. Dermatology 186, 23–31. doi: 10.1159/000247298, PMID: 8435513

[ref11] Furue M. (2020). Regulation of Filaggrin, Loricrin, and Involucrin by IL-4, IL-13, IL-17A, IL-22, AHR, and NRF2: pathogenic implications in atopic dermatitis. Int. J. Mol. Sci. 21:5382. doi: 10.3390/ijms21155382, PMID: 32751111 PMC7432778

[ref12] Han Y. Kim B. Ban J. Lee J. Kim B. J. Choi B. S.. (2012). A randomized trial of *Lactobacillus plantarum* CJLP133 for the treatment of atopic dermatitis. Pediatr. Allergy Immunol. 23, 667–673. doi: 10.1111/pai.12010, PMID: 23050557

[ref13] Hanifin J. M. Rajka G. (1980). Diagnostic features of atopic dermatitis. Acta Dermato Venereol 1980, 44–47.

[ref14] Howell M. D. Kim B. E. Gao P. Grant A. V. Boguniewicz M. DeBenedetto A.. (2009). Cytokine modulation of atopic dermatitis filaggrin skin expression. J. Allergy Clin. Immunol. 124, R7–R12. doi: 10.1016/j.jaci.2009.07.012 19720210 PMC13237620

[ref15] Irvine A. D. McLean W. H. Leung D. Y. (2011). Filaggrin mutations associated with skin and allergic diseases. N. Engl. J. Med. 365, 1315–1327. doi: 10.1056/NEJMra1011040 21991953

[ref16] Ishikawa J. Narita H. Kondo N. Hotta M. Takagi Y. Masukawa Y.. (2010). Changes in the ceramide profile of atopic dermatitis patients. J. Invest. Dermatol. 130, 2511–2514. doi: 10.1038/jid.2010.161, PMID: 20574438

[ref17] Janssens M. van Smeden J. Gooris G. S. Bras W. Portale G. Caspers P. J.. (2012). Increase in short-chain ceramides correlates with an altered lipid organization and decreased barrier function in atopic eczema patients. J. Lipid Res. 53, 2755–2766. doi: 10.1194/jlr.P030338, PMID: 23024286 PMC3494247

[ref18] Jeong K. Kim M. Jeon S. A. Kim Y. H. Lee S. (2020). A randomized trial of *Lactobacillus rhamnosus* IDCC 3201 tyndallizate (RHT3201) for treating atopic dermatitis. Pediatr. Allergy Immunol. 31, 783–792. doi: 10.1111/pai.13269, PMID: 32363613

[ref19] Kim J. H. Baek J. Sa S. Park J. Kih M. Kim W. (2021). Kestose-enriched fructo-oligosaccharide alleviates atopic dermatitis by modulating the gut microbiome and immune response. J. Funct. Foods 85:104650. doi: 10.1016/j.jff.2021.104650

[ref20] Kim S. Han S. Y. Lee J. Kim N. R. Lee B. R. Kim H.. (2022). *Bifidobacterium longum* and galactooligosaccharide improve skin barrier dysfunction and atopic dermatitis-like skin. Allergy Asthma Immunol Res. 14, 549–564. doi: 10.4168/aair.2022.14.5.549, PMID: 36174995 PMC9523416

[ref21] Kim J. Kim B. E. Berdyshev E. Bronova I. Bin L. Bae J.. (2023a). *Staphylococcus aureus* causes aberrant epidermal lipid composition and skin barrier dysfunction. Allergy 78, 1292–1306. doi: 10.1111/all.15640, PMID: 36609802 PMC12727050

[ref22] Kim J. Kim B. E. Goleva E. Berdyshev E. Bae J. Kim S.. (2023b). Alterations of epidermal lipid profiles and skin microbiome in children with atopic dermatitis. Allergy Asthma Immunol Res. 15, 186–200. doi: 10.4168/aair.2023.15.2.186, PMID: 37021505 PMC10079518

[ref23] Kim B. E. Leung D. Y. M. (2018). Significance of skin barrier dysfunction in atopic dermatitis. Allergy Asthma Immunol Res. 10, 207–215. doi: 10.4168/aair.2018.10.3.207, PMID: 29676067 PMC5911439

[ref24] Kim B. K. Shon J. C. Seo H. S. Liu K. H. Lee J. W. Ahn S. K.. (2022). Decrease of ceramides with long-chain fatty acids in psoriasis: possible inhibitory effect of interferon gamma on chain elongation. Exp. Dermatol. 31, 122–132. doi: 10.1111/exd.14431 34270128

[ref25] Knox S. O'Boyle N. M. (2021). Skin lipids in health and disease: a review. Chem. Phys. Lipids 236:105055. doi: 10.1016/j.chemphyslip.2021.105055, PMID: 33561467

[ref26] Koga Y. Tokunaga S. Nagano J. Sato F. Konishi K. Tochio T.. (2016). Age-associated effect of kestose on Faecalibacterium prausnitzii and symptoms in the atopic dermatitis infants. Pediatr. Res. 80, 844–851. doi: 10.1038/pr.2016.167, PMID: 27537603 PMC5156669

[ref27] Langan S. M. Irvine A. D. Weidinger S. (2020). Atopic dermatitis. Lancet 396, 345–360. doi: 10.1016/S0140-6736(20)31286-1 32738956

[ref28] Leung D. Y. M. Berdyshev E. Goleva E. (2020). Cutaneous barrier dysfunction in allergic diseases. J. Allergy Clin. Immunol. 145, 1485–1497. doi: 10.1016/j.jaci.2020.02.021, PMID: 32507227 PMC7291847

[ref29] Olesen C. M. Clausen M. L. Andersen P. S. Agner T. (2018). The skin microbiome in atopic dermatitis—a potential treatment target? Curr. Derm. Rep. 7, 199–208. doi: 10.1007/s13671-018-0245-6

[ref30] Opálka L. Kováčik A. Maixner J. Vávrová K. (2016). Omega-O-Acylceramides in skin lipid membranes: effects of concentration, Sphingoid Base, and model complexity on microstructure and permeability. Langmuir 32, 12894–12904. doi: 10.1021/acs.langmuir.6b03082, PMID: 27934529

[ref31] Passeron T. Lacour J. P. Fontas E. Ortonne J. P. (2006). Prebiotics and synbiotics: two promising approaches for the treatment of atopic dermatitis in children above 2 years. Allergy 61, 431–437. doi: 10.1111/j.1398-9995.2005.00956.x, PMID: 16512804

[ref32] Rostaher A. Rodriguez-Campos S. Deplazes P. Zwickl L. Akdis A. C. Urwyler A.. (2021). Atopic dermatitis in West Highland white terriers—part III: early life peripheral blood regulatory T cells are reduced in atopic dermatitis. Vet. Dermatol. 32, 239–e63. doi: 10.1111/vde.12939, PMID: 33565202

[ref33] Shibata R. Kimura M. Takahashi H. Mikami K. Aiba Y. Takeda H.. (2009). Clinical effects of kestose, a prebiotic oligosaccharide, on the treatment of atopic dermatitis in infants. Clin. Exp. Allergy 39, 1397–1403. doi: 10.1111/j.1365-2222.2009.03295.x, PMID: 19508323

[ref34] Ständer S. Yosipovitch G. Bushmakin A. G. Cappelleri J. C. Luger T. Tom W. L.. (2019). Examining the association between pruritus and quality of life in patients with atopic dermatitis treated with crisaborole. J. Eur. Acad. Dermatol. Venereol. 33, 1742–1746. doi: 10.1111/jdv.15712, PMID: 31132182

[ref35] Suzuki M. Ohno Y. Kihara A. (2022). Whole picture of human stratum corneum ceramides, including the chain-length diversity of long-chain bases. J. Lipid Res. 63:100235. doi: 10.1016/j.jlr.2022.100235, PMID: 35654151 PMC9240646

[ref36] Tochio T. Kadota Y. Tanaka T. Koga Y. (2018). 1-Kestose, the smallest Fructooligosaccharide component, which efficiently stimulates *Faecalibacterium prausnitzii* as well as Bifidobacteria in humans. Food Secur. 7:140. doi: 10.3390/foods7090140, PMID: 30200390 PMC6164784

[ref37] van den Bogaard E. H. Elias P. M. Goleva E. Berdyshev E. Smits J. P. H. Danby S. G.. (2023). Targeting skin barrier function in atopic dermatitis. J. Allergy Clin. Immunol. Pract. 11, 1335–1346. doi: 10.1016/j.jaip.2023.02.005 36805053 PMC11346348

[ref38] Westerberg R. Tvrdik P. Undén A. B. Månsson J. E. Norlén L. Jakobsson A.. (2004). Role for ELOVL3 and fatty acid chain length in development of hair and skin function. J. Biol. Chem. 279, 5621–5629. doi: 10.1074/jbc.M310529200, PMID: 14581464

[ref39] Yoon S. H. Ha S. M. Kwon S. Lim J. Kim Y. Seo H.. (2017). Introducing EzBioCloud: a taxonomically united database of 16S rRNA gene sequences and whole-genome assemblies. Int. J. Syst. Evol. Microbiol. 67, 1613–1617. doi: 10.1099/ijsem.0.001755, PMID: 28005526 PMC5563544

[ref40] Zheng Y. Wang Q. Ma L. Chen Y. Gao Y. Zhang G.. (2019). Alterations in the skin microbiome are associated with disease severity and treatment in the perioral zone of the skin of infants with atopic dermatitis. Eur. J. Clin. Microbiol. Infect. Dis. 38, 1677–1685. doi: 10.1007/s10096-019-03598-9, PMID: 31152265

[ref41] Zwara A. Wertheim-Tysarowska K. Mika A. (2021). Alterations of ultra long-chain fatty acids in hereditary skin diseases-review article. Front. Med. 8:730855. doi: 10.3389/fmed.2021.730855, PMID: 34497816 PMC8420999

