# Supplementary figures and images for: Advanced fructo-oligosaccharides improve itching and aberrant epidermal lipid composition in children with atopic dermatitis
Source: Front Microbiol. 2024 Apr 29;15:1383779. doi: 10.3389/fmicb.2024.1383779 (PMC11089124; doi:10.3389/fmicb.2024.1383779)

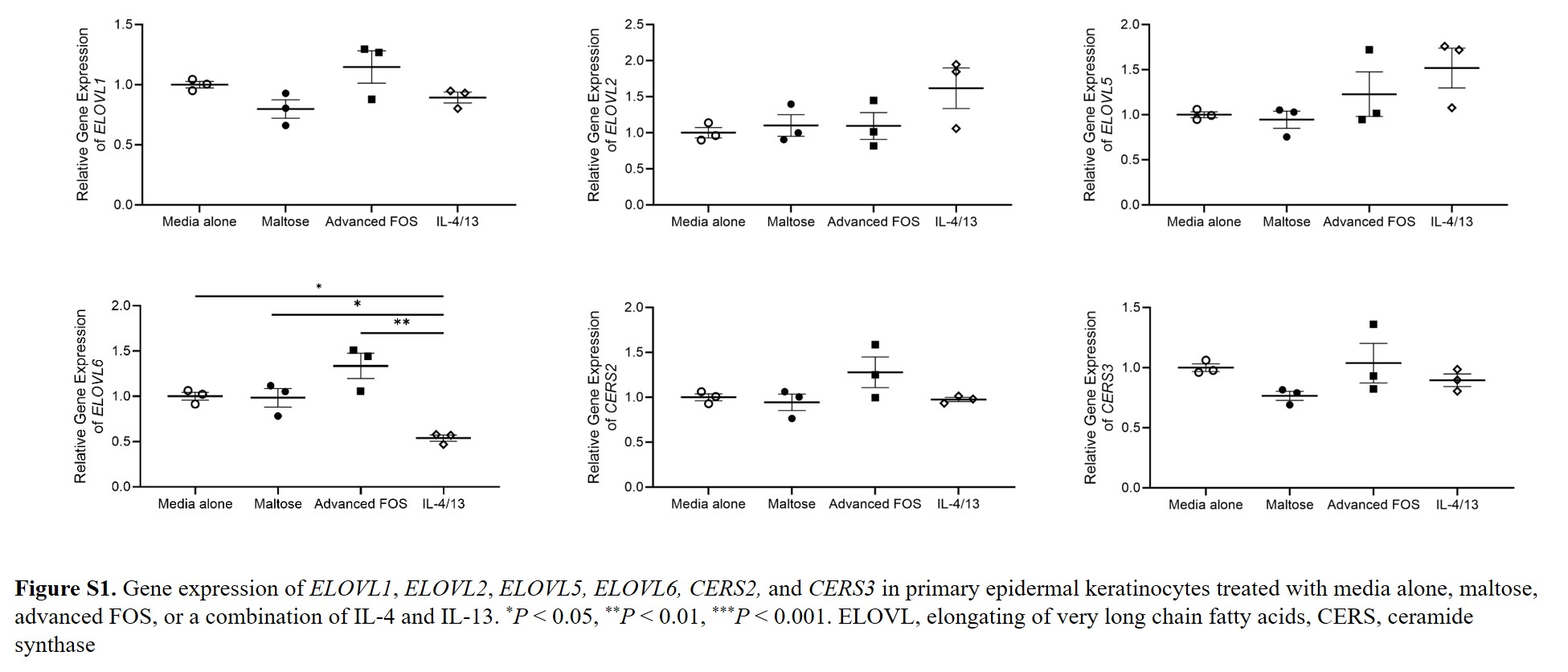

Supplement: Supplementary file 2 [file Image_1.JPEG]
